# *Ccl2*, *Cx3cr1* and *Ccl2*/*Cx3cr1* chemokine deficiencies are not sufficient to cause age-related retinal degeneration

**DOI:** 10.1016/j.exer.2012.11.015

**Published:** 2013-02

**Authors:** Ulrich F.O. Luhmann, Livia S. Carvalho, Scott J. Robbie, Jill A. Cowing, Yanai Duran, Peter M.G. Munro, James W.B. Bainbridge, Robin R. Ali

**Affiliations:** a Department of Genetics, UCL Institute of Ophthalmology, 11-43 Bath Street, EC1V9EL London, United Kingdom; b Imaging Unit, UCL Institute of Ophthalmology, London, United Kingdom; c NIHR Biomedical Research Centre for Ophthalmology, London, United Kingdom

**Keywords:** chemokine knockout mice, Ccl2/Cx3cr1 double knockout mice, Ccl2, Cx3cr1, age-related macular degeneration, retinal degeneration, subretinal macrophages, genetic background

## Abstract

Monocytes, macrophages, dendritic cells and microglia play critical roles in the local immune response to acute and chronic tissue injury and have been implicated in the pathogenesis of age-related macular degeneration. Defects in *Ccl2-Ccr2* and *Cx3cl1-Cx3cr1* chemokine signalling cause enhanced accumulation of bloated subretinal microglia/macrophages in senescent mice and this phenomenon is reported to result in the acceleration of age-related retinal degeneration. The purpose of this study was to determine whether defects in CCL2-CCR2 and CX3CL1-CX3CR1 signalling pathways, alone or in combination, cause age-dependent retinal degeneration. We tested whether three chemokine knockout mouse lines, *Ccl2*^*−/−*^, *Cx3cr1*^*−/−*^ and *Ccl2*^*−/−*^*/Cx3cr1*^*−/−*^, in comparison to age-matched *C57Bl/6* control mice show differences in subretinal macrophage accumulation and loss of adjacent photoreceptor cells at 12–14 months of age. All mouse lines are derived from common parental strains and do not carry the homozygous *rd8* mutation in the *Crb1* gene that has been a major confounding factor in previous reports. We quantified subretinal macrophages by counting autofluorescent lesions in fundus images obtained by scanning laser ophthalmoscopy (AF-SLO) and by immunohistochemistry for Iba1 positive cells. The accumulation of subretinal macrophages was enhanced in *Ccl2*^*−/−*^, but not in *Cx3cr1*^*−/−*^ or *Ccl2*^*−/−*^*/Cx3cr1*^*−/−*^ mice. We identified no evidence of retinal degeneration in any of these mouse lines by TUNEL staining or semithin histology. In conclusion, CCL2-CCR2 and/or CX3CL1-CX3CR1 signalling defects may differentially affect the trafficking of microglia and macrophages in the retina during ageing, but do not appear to cause age-related retinal degeneration in mice.

## Highlights

► No increase in subretinal macrophages in aged *Cx3cr1*^−/−^ and *Ccl2*^−/−^/*Cx3cr1*^−/−^ mice. ► Increase in subretinal macrophages in *Ccl2*^−/−^ mice at 12–14 months of age. ► No age-related retinal degeneration in *Ccl2*^*−/−*^, *Cx3cr1*^−/−^ or *Ccl2*^−/−^/*Cx3cr1*^−/−^ mice.

## 1 Introduction

Monocytes, macrophages, dendritic cells, and microglia are key regulator and effector cells of the immune system and have specific roles in immune surveillance and maintenance of tissue homeostasis (Geissmann et al., 2010).

Monocytes circulate between the bone marrow, blood and spleen and consist of several phenotypic and functional subsets (Geissmann et al., 2003). These monocyte subsets can be distinguished by the expression pattern of chemokine surface receptors and adhesion molecules, that include CD14, CD16 and CD64 (in human) and CD115, CD11b, Gr1/Ly6C (in mouse) as well as the C–C chemokine receptor 2 (CCR2) and fractalkine receptor CX3CR1 (in both) (Tacke and Randolph, 2006). These molecules also determine a particular mode of trafficking and functional behaviour of the respective subsets (Geissmann et al., 2003; Jung et al., 2000). Pro-inflammatory/classical CCR2-expressing monocytes (human: CD14^+^, CD16^−^CD64^+^; mouse: CD11b^+^, Ly6C^hi^) infiltrate sites of inflammation and give rise to inflammatory macrophages and dendritic cells in response to local stimuli such as the inducible CC-chemokine ligand 2 (Ccl2) (Boring et al., 1997; Dzenko et al., 2005; Lu et al., 1998; Randolph and Furie, 1995). In contrast “resting”/non-classical monocytes, which express high levels of Cx3cr1, but not CCR2 (human: CD14^lo^, CD16^+^CD64^−^; mouse: CD11b^+^, Ly6C^low^), patrol non-inflamed tissue and may be involved in the resolution of inflammatory processes (Auffray et al., 2007; Geissmann et al., 2003; Nahrendorf et al., 2007; Tacke and Randolph, 2006; Yona and Jung, 2010).

Tissue resident macrophages and dendritic cells are present in virtually all healthy tissues of the body. They form a network of cells engaged in local immune surveillance and tissue homeostasis either through the phagocytosis of metabolic by-products, apoptotic cells and pathogens or through the controlled production of cytokines and growth factors (Auffray et al., 2009; Geissmann et al., 2010; McMenamin, 1999). Microglia are the tissue resident immune cells of the central nervous system (CNS) and may be located adjacent to vessels as perivascular microglia or form a parenchymal network, which in the retina is found at the level of the inner (IPL) and outer plexiform layers (OPL) (Xu et al., 2009). Microglia express high levels of Cx3cr1 (Jung et al., 2000) and under physiological conditions show a continuous surveillance behaviour with highly dynamic movements of their processes but a stable soma position (Nimmerjahn et al., 2005). In response to injury, infections or any other disturbance of tissue homeostasis, including retinal degenerations, microglia become activated and alter their morphology from a ramified to an amoeboid appearance, whilst at the same time migrating to the site of tissue injury (Eter et al., 2008; Liang et al., 2009; Luhmann et al., 2012; Paques et al., 2010).

The dynamics of resting microglia engaged in surveillance and their re-distribution in response to activation (such as by acute injury, excessive light exposure or ageing) are thought to be controlled by the two chemokine signalling pathways *Ccl2-Ccr2* and *Cx3cl1-Cx3cr1* (Combadiere et al., 2007; Liang et al., 2009; Luhmann et al., 2009; Nakazawa et al., 2007; Ng and Streilein, 2001). A functional role of these two pathways for the maintenance of tissue homeostasis and the control of associated myeloid cell activation during normal ageing and in age-related retinal disease has been suggested by the observations that CX3CR1 expression in human non-classical monocytes decreases with normal ageing, whilst plasma levels of CCL2 increase (Seidler et al., 2010). Furthermore, aqueous samples from eyes with advanced exudative age-related macular degeneration contain higher levels of CCL2 compared with age-matched controls and CCL2 is increased with normal ageing in the RPE/choroidal complex of aged C57Bl/6 mice (Chen et al., 2008). These findings suggest that ageing and chronic inflammatory conditions are associated with alterations in chemokine signalling. Further support for this comes from the observation that *Cx3cr1*^−/−^ (Chinnery et al., 2012; Combadiere et al., 2007; Raoul et al., 2008) and *Ccl2*^−/−^ mice (Luhmann et al., 2009) develop an accelerated age-related accumulation of bloated subretinal macrophages or microglia in mice older than 18 months, a process that also occurs in wildtype mice during ageing, though to a lesser extent (Xu et al., 2007). In the first report regarding the phenotype of *Ccl2*^−/−^ and *Ccr2*^−/−^ knockout mice, these subretinal macrophages were misinterpreted as drusen-like lesions, as a result of similarities in their fundal appearance (Ambati et al., 2003). Immunohistological examination demonstrates that these lesions are not sub-RPE deposits, but subretinal macrophages that are bloated by the accumulation of lipofuscin with age (Luhmann et al., 2009; Xu et al., 2007) and are consequently evident on scanning laser ophthalmoscopy (SLO) as discrete autofluorescent spots.

There are conflicting reports as to whether the accumulation of dysfunctional subretinal macrophages results in retinal degeneration (Ambati et al., 2003; Chen et al., 2011; Chinnery et al., 2012; Combadiere et al., 2007; Luhmann et al., 2009; Ng and Streilein, 2001). While one group has reported that both *Ccl2* and *Ccr2* knockout mouse lines develop RPE and choroidal pathology from the age of 9 months onwards leading to subsequent retinal pathology around 16 months of age (Ambati et al., 2003), another group claims that pronounced RPE lesions only arise in mice older than 18 months of age and even then only with variable penetrance (40% penetrance in *Ccl2* and 24% in *Ccr2* knockout mice) (Chen et al., 2011). In contrast, we have previously only observed normal age-related changes in *Ccl2*^−/−^, similar to those seen in age-matched C57Bl/6 wildtype mice up to an age of 27 months despite a clearly increased accumulation of subretinal macrophages in senescent mice (Luhmann et al., 2009). Similar conflicting reports exist for *Cx3cr1*^−/−^ mice, with one study attesting to the development of age-related retinal degeneration in *Cx3cr1*^−/−^ mice (Combadiere et al., 2007) and another more recent report suggesting that this degeneration is not due to the deletion in *Cx3cr1*, but to discrepancies in the genetic background of the mouse line (Chinnery et al., 2012). Differences have also been attributed to the nature of environmental housing conditions where variations in light and pathogen exposure might alter the activation state of microglia or systemically derived macrophages thereby enhancing or attenuating the retinal pathology seen in different mouse lines.

In a recent study of CCDKO (*Ccl2*^−/−^/*Cx3cr1*^−/−^) mice that were backcrossed to generate re-derived, closely related *Ccl2*^−/−^, *Cx3cr1*^−/−^ and *Ccl2*^−/−^/*Cx3cr1*^−/−^ mouse lines, we identified the *rd8* mutation in the *Crb1* gene as a dominant confounding factor that was responsible for the early onset retinal degeneration previously attributed to the combined deficiency of *Ccl2* and *Cx3cr1* in CCDKO mice (Luhmann et al., 2012; Tuo et al., 2007). In contrast to many other lines that carry the Cx3cr1-GFP reporter allele (Jung et al., 2000), these mouse lines do not carry this or any other reporter alleles. The identification of the confounding *rd8* mutation not only in the CCDKO mouse strain, but also in many other mouse strains with a *C57Bl/6N* background (Mattapallil et al., 2012) raises the question, whether the previously reported phenotypes of different chemokine single knockout mice might also have been affected by the rd8 mutation. In this study we therefore compare the newly established (*rd8* mutation-free) chemokine knockout mouse lines to investigate whether individual or combined defects in *Ccl2* and/or *Cx3cr1* signalling result in retinal degeneration with age.

## 2 Materials and methods

### 2.1 Animals and housing conditions

Animals for these experiments were obtained from *Ccl2*^−/−^, *Cx3cr1*^−/−^ single and *Ccl2*^−/−^/*Cx3cr1*^−/−^ double knockout mouse lines at the age of 12–14 months, which are all derived from our previously described backcross experiments of the *CCDKO* line (*Ccl2*^−/−^/*Cx3cr1*^−/−^/*Crb1*^*rd8/rd8*^) with *C57Bl/6J Ola Hsd* mice (Luhmann et al., 2012). The original parental line *CCDKO* was originally described by Tuo et al. and was kindly provided by Chi Chao Chan (Tuo et al., 2007).

Genotyping for the two chemokine loci was performed by PCR, while sequencing was used to evaluate the *rd8* genotype as described recently (Luhmann et al., 2012; Mattapallil et al., 2012). These experiments confirmed the respective chemokine genotypes and verified that all analysed mice in this study did not carry the *rd8* mutation in a homozygous state, but were either heterozygous (*Crb1*^*+/rd8*^) or wildtype (*Crb1*^*+/+*^) for the *rd8* mutation in the *Crb1* gene.

All animals were housed in the same room under a 12 h/12 h dark light cycle with a mean luminescence during the light period at the bottom of the cage of 33 ± 28 lx. Furthermore, the animals have access to cover (e.g. paper roll and excess of bedding) inside the cage which allows them to burrow. Control animals were age-matched *C57Bl/6J OLA Hsd* mice (Harlan UK Ltd., Blackthorn, UK) that were imported as young adult mice at 6–8 weeks of age and housed in the same room next to the experimental mice.

For *in vivo* SLO imaging, mice were anesthetized by a single intraperitoneal (IP) injection of a mixture of medetomidine hydrochloride (1 mg/kg body weight; Domitor; Pfizer Animal Health, New York, NY), and ketamine (60 mg/kg body weight) in water. All pupils were dilated with 1 drop of 1% tropicamide. The animal experiments were performed in accordance with the ARVO Statement for the Use of Animals in Ophthalmic and Vision Research, the EU Directive 2010/63/EU for animal experiments and under the UK Home Office project licence (PPL 70/1279).

### 2.2 Autofluorescence imaging and phenotyping of mice using scanning laser ophthalmoscopy

Autofluorescence imaging was performed using a HRA2 scanning laser ophthalmoscope with a 55° angle lens as described previously (Luhmann et al., 2009). We used the autofluorescent mode of the HRA2 (Heidelberg engineering, Heidelberg, Germany) to scan the retina with a 488 nm laser that provides the excitation light to stimulate emission of autofluorescence from any possible fluorophores in the retina including e.g. lipofuscin in the RPE. The optic disc was positioned at the centre of the image and the image focused either on the inner retinal vasculature, the inner retinal layer or the outer retina, respectively. Projection images of 30 frames per fundus were taken and evaluated for the appearance of autofluorescence. Quantitative assessment of AF-SLO images was performed by counting the number of discrete autofluorescent spots per fundus image in the autofluorescent images for each animal after these had been processed in Adobe Photoshop CS 2 (Adobe Systems Incorporated, UK) using the auto level function. The number of autofluorescent spots per fundus image and animal was calculated by averaging the data obtained from the left and the right eye.

### 2.3 Immunohistochemistry, tunnel staining and cell counts

Animals were terminally anaesthetised with pentobarbital (Euthatal, Merial, UK) and subjected to cardiac perfusion with 0.8% paraformaldehyde (PFA)/PBS. Eyes were then embedded in OCT media and sectioned. All eyes were sequentially sectioned across five slides to provide a representative cross-section of the eye with around 30 sections per each individual slide. Separate slides were then used either for microglia staining or for TUNEL detection.

For microglia labelling, retinal sections were post-fixed with 4% PFA/PBS for 10 min and blocked with PBS/1% BSA (Sigma Aldrich, Steinheim, Germany)/5% nonspecific goat serum (AbD Serotec, Kidlington, UK) including 0.3% Triton X-100 for permeabilisation for 1 h followed by overnight incubation at 4 °C with a 1:200 dilution of anti Iba1 antibody (final concentration = 2.5 ug/ml, Code No. 019-19741, WAKO, Osaka, Japan) in blocking solution. The next day sections were washed 3–4 times with 1× PBS and incubated for 2 h with a 1:500 dilution of goat anti-rabbit AlexaFluor 488 nm-conjugated secondary antibody (final concentration 4 μg/ml, #A11034; Invitrogen-Molecular Probes, Leiden, The Netherlands) and washed again 3 times with 1× PBS.

TUNEL staining was performed in retinal section from animals perfused with 0.8% PFA/PBS and followed the protocol described by the manufacturers in the ApopTag^®^ Red *In Situ* Apoptosis Detection Kit (Chemicon International Inc, Merck Millipore, USA).

After staining retinal sections were mounted with fluorescence mounting medium containing Hoechst 33342 (Dako, Cambridgeshire, UK) and images were obtained using a confocal laser scanning microscope (Leica DM5500 Q, Leica Microsystems, Wetzlar, Germany).

Cell counts were performed masked using a light fluorescent microscope (Observer.Z1 Axio, Carl Zeiss Microimaging, Jena, Germany). Counts were obtained from 10 representative superior-inferior orientated sagittal retinal sections that were around 225 μm apart and distributed equally across the whole eye. Only TUNEL positive cells found in the outer nuclear layer or Iba1 positive cells between the outer segment area and the RPE were counted. Iba-1 positive processes that did not also display a Hoechst-positive nucleus were not counted as positive events.

### 2.4 Histopathology of the retina

Semithin histological morphometric analyses were performed as described previously (Luhmann et al., 2009). Animals were sacrificed and cardiac perfusion with 1% PFA performed before the eyes were enucleated and fixed in 3% glutaraldehyde and 1% paraformaldehyde in 0.08 M sodium cacodylate-HCl (pH 7.4) for at least 30 h at 4 °C. The cornea and lens were removed and the eye cups oriented and post-fixed in 1% aqueous osmium tetroxide for 2 h, dehydrated by an ascending ethanol series (50%–100%) and propylene oxide, and infiltrated overnight with a 1:1 mixture of propylene oxide and araldite resin (Agar Scientific Limited, Essex UK). After 8 h in full resin, the eyes were embedded in fresh resin and incubated overnight at 60 °C. Semithin (0.7 μm) sections were cut in the inferior–superior axis passing through the optic nerve head with a microtome (Ultracut S; Leica, Wetzlar, Germany). Sections were then stained with a 1% mixture of toluidine blue-borax in 50% ethanol and images taken using bright field microscopy (Observer.Z1 Axio, Carl Zeiss Microimaging, Jena, Germany). For quantitative analysis, images were imported into Image J and the rows of photoreceptors counted using the counting tool (Schneider et al., 2012).

### 2.5 Statistical analysis

Statistical analyses were performed using GraphPad Prism 5 for Windows (GraphPad Software Inc, La Jolla, USA).

## 3 Results

### 3.1 Unlike *Ccl2*^−/−^ mice, *Cx3cr1*^−/−^ and *Ccl2*^−/−^/*Cx3cr1*^−/−^ mice do not exhibit an increased accumulation of subretinal macrophages at 12–14 month of age

Autofluorescent scanning laser ophthalmoscopy (AF-SLO) was used to determine whether the three closely related *Ccl2*^−/−^, *Cx3cr1*^−/−^ and *Ccl2*^−/−^*/Cx3cr1*^−/−^ chemokine knockout mouse lines, that do not carry the homozygous *rd8* mutation, develop a more pronounced age-related accumulation of subretinal macrophages than *C57Bl/6* control mice.

**Fig. 1:**
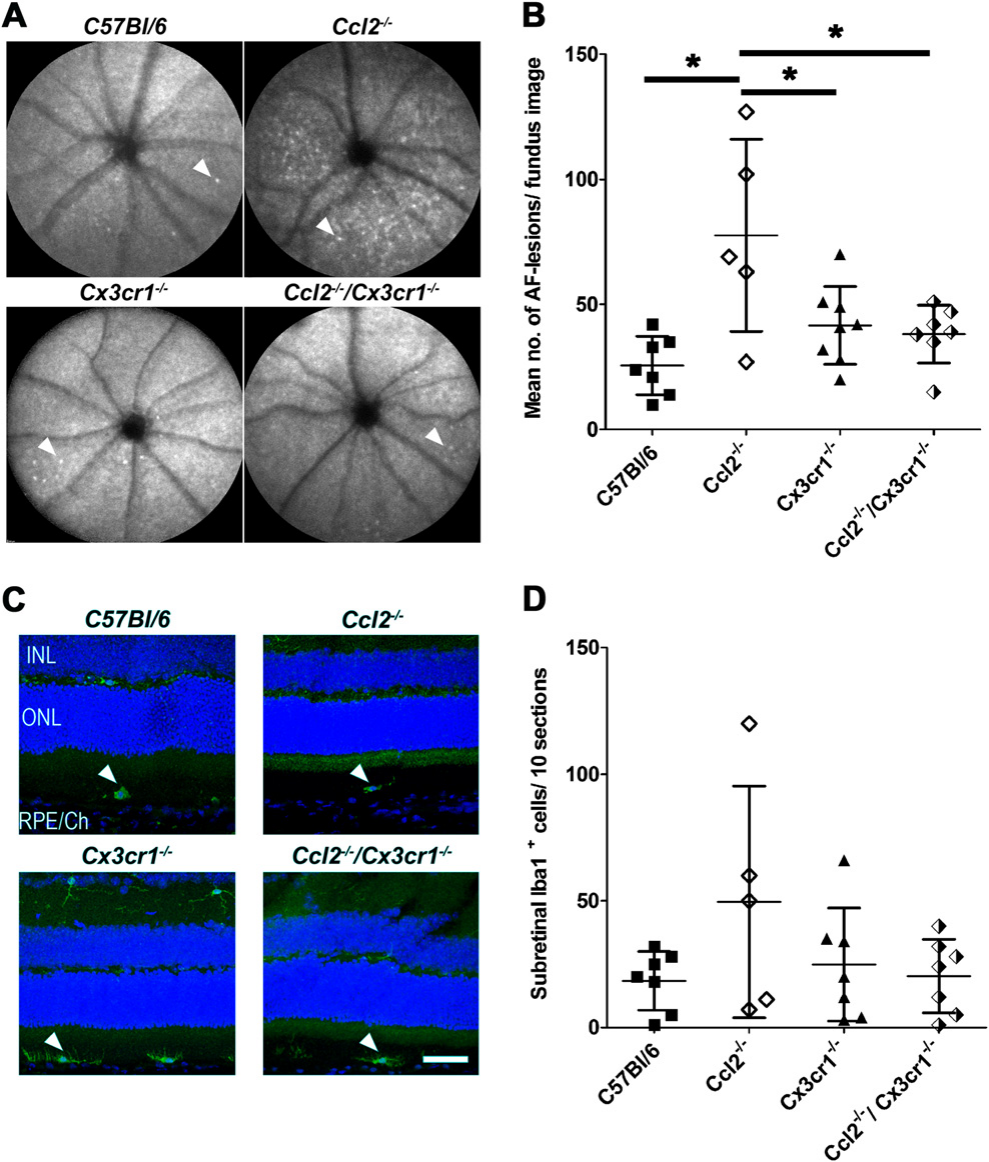
A. Autofluorescent scanning laser ophthalmoscopy fundus images (AF-SLO) of C57Bl/6, *Ccl2*^−/−^, *Cx3cr1*^*−/−*^ and *Ccl2*^*−/−*^*/Cx3cr1*^*−/−*^ knockout mice. Images are focused on the outer retina and reveal discrete autofluorescent lesions (white arrowheads) indicative of subretinal macrophages in all genotypes at the age of 12–14 months. B. Quantification of discrete autofluorescent spots in AF-SLO fundus images. The individual data points in this graph represent the mean number of autofluorescent spots between the left and the right eye of each animal (*n*). The mean number of autofluorescent spots ± standard deviation for each group (genotype) is indicated by a small horizontal line and the error bars. *Ccl2*^*−/−*^ mice show significantly higher numbers of autofluorescent spots per fundus image than the other two chemokine deficient lines or the *C57Bl/6* age-matched control. Stars indicate statistically significant changes (*p* = 0.0017, Oneway ANOVA with Tukey's multiple comparison *p* < 0.05; *n* (*C57Bl/6*) = 7, *n* (*Ccl2*^*−/−*^) = 5, *n* (*Cx3cr1*^*−/−*^) = 8, *n* (*Ccl2*^*−/−*^*/Cx3cr1*^*−/−*^) = 7). C. Immunohistochemistry for the macrophage/microglia marker Iba1 (green) on sagittal, superior-inferior oriented retinal sections obtained from 12 to 14 months old *C57Bl/6*, *Ccl2*^*−/−*^, *Cx3cr1*^*−/−*^ and *Ccl2*^*−/−*^*/Cx3cr1*^*−/−*^ knockout mice. White arrowheads indicate the position of Iba1 positive subretinal macrophages. Sections were counterstained with DAPI (blue) to label the nuclei. INL: Inner nuclear layer, ONL: Outer nuclear layer, RPE/Ch: retinal pigment epithelium and choroid. Scale bar: 50 μm. D. Quantification of Iba1 positive macrophages (white arrowheads, Fig. 1C) in the subretinal space of *C57Bl/6*, *Ccl2*^*−/−*^, *Cx3cr1*^*−/−*^ and *Ccl2*^*−/−*^*/Cx3cr1*^*−/−*^ mice. Subretinal macrophages were counted in 10 superior to inferior oriented sagittal sections that were about 225 μm apart and equally distributed across the eyes. One eye per animal was evaluated and the results for each group are shown as the sum of Iba1^+^ macrophages/10 sections ± standard deviation. The pattern observed is consistent with that in the AF-SLO counts (Fig. 1B), although no statistically significant differences between any of the four mouse lines were detected (*p* = 0.1653 Oneway ANOVA with Tukey's multiple comparison *p* < 0.05; *n* (*C57Bl/6*) = 7, *n* (*Ccl2*^*−/−*^) = 5, *n* (*Cx3cr1*^*−/−*^) = 7, *n* (*Ccl2*^*−/−*^*/Cx3cr1*^*−/−*^) = 7).

AF-SLO fundus images obtained from 12 to 14 months old mice from each genotype were similar in appearance and did not show the recently reported prominent autofluorescence signal seen in the inferior fundus of mice carrying a homozygous *rd8* mutation (Luhmann et al., 2012). AF-SLO images focused on the outer retina revealed discrete autofluorescent spots in all four genotypes (Fig. 1A, white arrowheads). These autofluorescent spots were not evenly distributed across the fundus image but were either seen as individual spots or localised in clusters (Fig. 1A). We have shown previously that these discrete autofluorescent spots are derived from bloated, lipofuscin-containing macrophages in the subretinal space (Luhmann et al., 2009). By quantifying these autofluorescent signals, we found that by the age of 12–14 months, C57Bl/6 mice have accrued 26 ± 12 (mean ± standard deviation) autofluorescent spots per fundus image, whilst *Ccl2*^−/−^ single knockout mice show 78 ± 38; *Cx3cr1*^−/−^ single knockout mice 41 ± 16 and *Ccl2*^−/−^/*Cx3cr1*^−/−^ mice 38 ± 12 autofluorescent spots. While in comparison to aged-matched control C57Bl/6 mice and both other chemokine knockout mouse lines this marker for subretinal macrophage accumulation showed a significant increase in *Ccl2*^−/−^ mice, no significant increase was observed in these newly established *Cx3cr1*^−/−^ and *Ccl2*^−/−^/*Cx3cr1*^−/−^ knockout mouse lines compared to C57B/l6 mice at 12–14 months of age (Oneway ANOVA with Tukey's multiple comparison test *p* < 0.05 (Fig. 1B).

To further evaluate and quantify the accumulation of subretinal macrophages/microglia in all four studied lines at this age, sagittal superior- to inferior-oriented retinal cryo-sections that were stained by immunohistochemistry against the macrophage/microglia marker Iba-1 (ionized calcium binding adaptor molecule 1, Fig. 1C, white arrowheads). This immunohistochemistry confirmed the presence of Iba-1 positive cells in the subretinal space of mice from all four genotypes (Fig. 1C, arrowheads). Quantification of Iba-1 positive subretinal cells across 10 representative and evenly distributed (about 225 μm apart) sections from whole eyes revealed an average of 18 ± 12 subretinal cells per 10 sections in C57Bl/6 mice; 50 ± 46 in *Ccl2*^−/−^ mice; 25 ± 22 cells in *Cx3cr1*^−/−^mice and 20 ± 15 cells in *Ccl2*^−/−^/*Cx3cr1*^−/−^ mice. Differences in the numbers observed between the various genotypes failed to achieve significance using this method (one way ANOVA with Tukey's multiple comparison *p* = 0.1653), although a trend in keeping with the results of AF-SLO lesion counts was observed (Fig. 1B). Very similar data were also obtained from counts of subretinal macrophages on semithin sections derived from the same animals (data not shown). All three data sets suggest that the absence of Cx3cr1 signalling, alone or in combination with a Ccl2 deficiency, does not result in more pronounced accumulation of subretinal macrophages.

### 3.2 *Ccl2*^−/−^, *Cx3cr1*^−/−^ and *Ccl2*^−/−^/*Cx3cr1*^−/−^ chemokine knockout mice show no signs of retinal degeneration or photoreceptor loss at 12–14 months of age

**Fig. 2:**
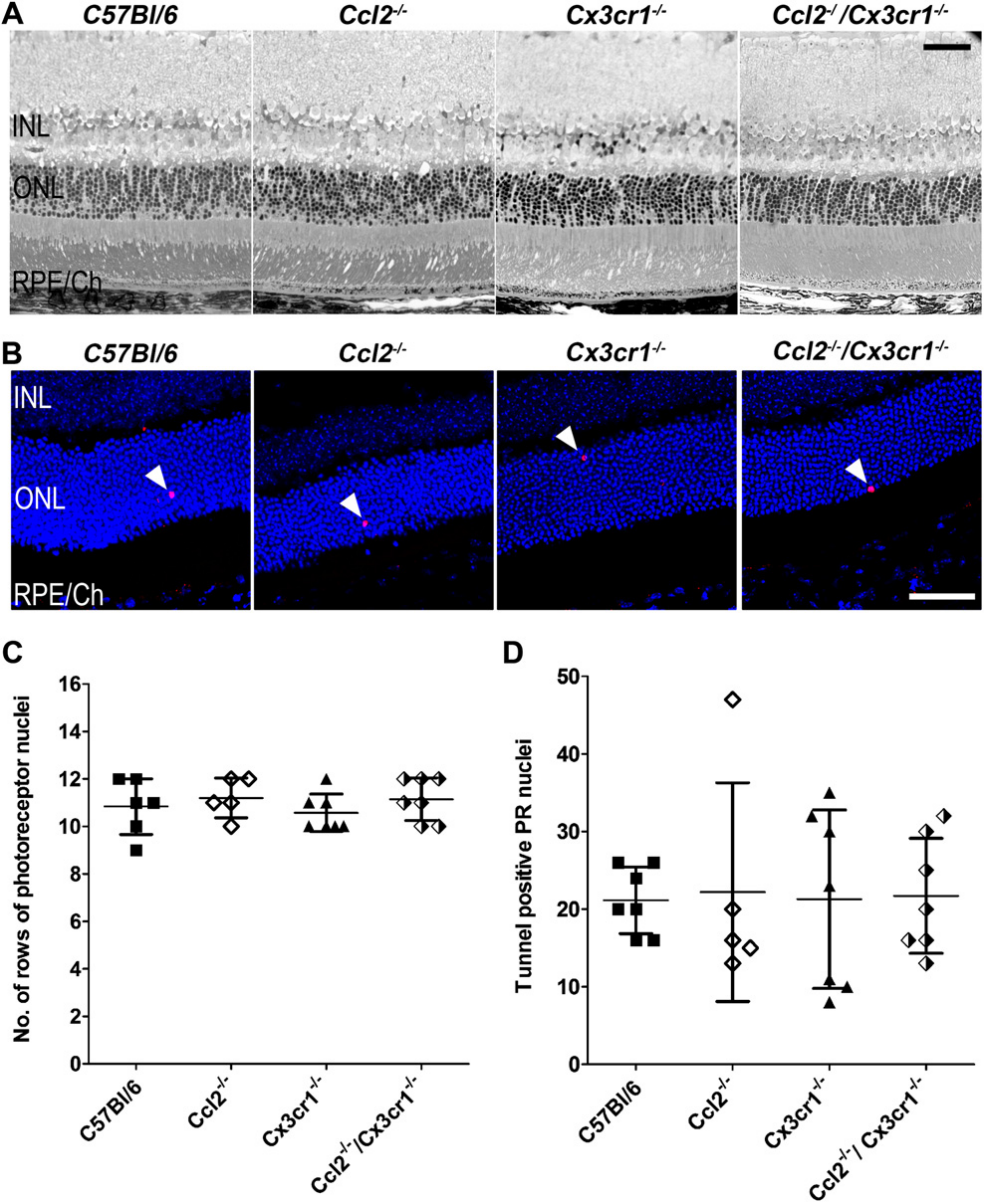
A. Semithin histology from the central retina of *C57Bl/6*, *Ccl2*^*−/−*^, *Cx3cr1*^*−/−*^ and *Ccl2*^*−/−*^*/Cx3cr1*^*−/−*^ mice. No gross alterations in retinal structure or RPE morphology were observed. B. Assessment of photoreceptor death by using TUNEL staining. Independent of the chemokine genotypes, all animals showed occasional TUNEL positive nuclei (white arrowheads) in the outer nuclear layer indicating a similar basal level of photoreceptor apoptosis at the age of 12–14 months. C. Quantification of photoreceptor nuclei in the outer nuclear layer (ONL) on semithin histological sections. We determined the number of rows of nuclei in the outer nuclear layer of all 4 mouse lines and did not observe statistically significant differences. All lines showed mean ± SD of 11 ± 1 rows of photoreceptor nuclei in the ONL, independent of the respective genotype (*p* = 0.6045 Oneway ANOVA with Tukey's multiple comparison *p* < 0.05, *n* (*C57Bl/6*) = 6, *n* (*Ccl2*^*−/−*^) = 5, *n* (*Cx3cr1*^*−/−*^) = 7, *n* (*Ccl2*^*−/−*^*/Cx3cr1*^*−/−*^) = 7). D. Quantification of TUNEL positive photoreceptor nuclei in the outer nuclear layer of *C57Bl/6*, *Ccl2*^*−/−*^, *Cx3cr1*^*−/−*^ and *Ccl2*^*−/−*^*/Cx3cr1*^*−/−*^ mice. 10 superior to inferior oriented sagittal retinal sections that were about 225 μm apart and equally distributed across the eye were evaluated and the sum of TUNEL positive photoreceptor nuclei in the outer nuclear layer for each animal are shown as individual data points. The mean ± standard deviation for each group is also indicated. No significant differences were observed between any of the three chemokine deficient mice as well as in comparison to C57Bl/6 mice (*p* = 0.9976 Oneway ANOVA with Tukey's multiple comparison *p* < 0.05, *n* (*C57Bl/6*) = 7, *n* (*Ccl2*^*−/−*^) = 5, *n* (*Cx3cr1*^*−/−*^) = 7, *n* (*Ccl2*^*−/−*^*/Cx3cr1*^*−/−*^) = 7). INL: inner nuclear layer, ONL: outer nuclear layer, RPE/Ch: retinal pigment epithelium/Choroid; scale bars 50 μm.

To determine whether the genetic defects in CCL2 and/or CX3CR1 signalling pathway(s) cause or affect the onset of retinal degeneration at the age of 12–14 months, we evaluated retinal morphology on semithin sections derived from each of the chemokine genotypes with that of age-matched C57Bl/6 mice at this time point. Consistent with the *in vivo* imaging results (Fig. 1A), gross retinal morphology was observed to be normal across all genotypes (Fig. 2A). To more specifically assess whether aged and dysfunctional macrophages in the subretinal space might have an effect on photoreceptor survival, we stained retinal sections for dying photoreceptors using TUNEL staining (Fig. 2B). Quantitative data for central retina photoreceptor rows count from semithin sections and TUNEL positive photoreceptor nuclei in the outer nuclear layer are shown in Fig. 2C and D, respectively. All genotypes, including wildtype mice, were found to have 11 ± 1 rows of photoreceptors in the outer nuclear layer of the central retina at 12–14 months of age (Fig. 2C). Basal levels of photoreceptor apoptosis, as indicated by TUNEL positive cell counts, were also similar across genotypes (TUNEL positive photoreceptor nuclei in C57Bl/6: 21 ± 4; *Ccl2*^−/−^ mice: 22 ± 14; *Cx3cr1*^−/−^: 21 ± 12 and *Ccl2*^−/−^/*Cx3cr1*^−/−^ mice: 22 ± 7 cells). Since neither of the techniques employed to determine retinal degeneration revealed any differences between the chemokine/chemokine receptor knockout mice or age-matched wildtype controls we conclude that dysfunctional chemokine signalling does not affect retinal photoreceptor survival up to the age of 12–14 months.

## 4 Discussion

The key findings of this study are that 12–14 months old *Cx3cr1*^−/−^ single and *Ccl2*^−/−^/*Cx3cr1*^−/−^ double knockout mice do not exhibit an increased accumulation of subretinal macrophages compared to age-matched C57Bl/6 mice, while *Ccl2*^−/−^ mice appear to manifest this phenotype at this age. Furthermore, none of these chemokine mouse lines have shown any evidence for degenerative changes in the retina either by *in vivo* autofluorescent fundus imaging, semithin histological analyses or by TUNEL labelling of apoptotic photoreceptors. This suggests that during normal ageing individual or combined genetic defects in CCL2 or CX3CR1 signalling alone are not sufficient to drive photoreceptor loss and age-related retinal degeneration above basal levels.

### 4.1 Chemokine signalling differentially affects age-related accumulation of subretinal macrophages

The observed increase in accumulation of subretinal macrophages in *Ccl2*^−/−^ mice at 12–14 months shows a high variability. This may indicate that the phenotype may not yet robustly be manifested in all mice of this group at the studied age. Such an interpretation of the data may also explain why both employed techniques show a similar pattern for the accumulation of subretinal macrophages which only reach statistical significance by *in vivo* AF-SLO analysis. Despite these uncertainties regarding the statistical robustness of the two data sets, the increase in accumulation of subretinal macrophages in this *Ccl2* line would be consistent with results from a separate study involving a different *Ccl2*^−/−^ knockout mouse line that showed a pronounced age-related-accumulation of subretinal macrophages at 20–24 months of age (Luhmann et al., 2009) supporting the conclusion that Ccl2 deficiency significantly affects macrophage/microglia trafficking to or from the subretinal space.

More important though is that in our study *Cx3cr1*^−/−^ deficient mice up to an age of 12–14 months did not show any indication for an accelerated accumulation of subretinal macrophages, even though this phenotype has been reported to be a prominent feature of *Cx3cr1*^−/−^ mice at 18 months and even at 2–3 months on both C57Bl/6 and Balb/c genetic backgrounds (Chinnery et al., 2012; Combadiere et al., 2007; Raoul et al., 2008). Our findings at the age of 12–14 months therefore seem to contradict these previous reports and questions the extent to which defective *Cx3cr1* chemokine signalling influences the normal age-related accumulation of subretinal microglia (Xu et al., 2007).

Since *Ccl2*^−/−^/*Cx3cr1*^−/−^ mice showed similar levels of subretinal microglia/macrophages as *Cx3cr1*^−/−^ and C57Bl/6 mice, CX3CR1 deficiency may affect the migratory capacity of macrophages and/or microglia in such a way as to abrogate the phenotype observed with CCL2 deficiency alone. This finding may support an interaction of these two chemokine pathways for modulating the migratory capacity of microglia and macrophages. *In vitro* studies with human monocytes expressing different variants of CX3CR1 (M280T) also suggest such an interaction (Combadiere et al., 2007).

### 4.2 CCL2 and/or CX3CR1 chemokine deficiencies are not sufficient to cause age-related retinal degeneration

Our data reveals that the combined genetic deficiencies of CCL2 and CX3CR1 as well as the individual deficiencies are not sufficient to induce age-related retinal degeneration up to 14 months. We cannot exclude the possibility that retinal degeneration might have occurred in our chemokine deficient strains with additional ageing. However, in a separate study involving a different *Ccl2*^−/−^ knockout mouse line there was no retinal degeneration in mice aged up to 25 months (Luhmann et al., 2009).

### 4.3 Discrepancies between different studies may be explained by additional genetic and/or environmental factors

Our findings are in clear contrast to several reports that describe an age-related retinal degeneration in other chemokine knockout mouse lines. For *Ccl2*^−/−^ and *Ccr2*^−/−^ knockout mice, one study has reported that RPE and choroidal changes occur from an age of about 9 months and are followed by subsequent retinal degeneration around 16 months of age (Ambati et al., 2003), while a second report describes the development of specific RPE lesions with a variable penetrance of 40% in *Ccl2*^−/−^ and 25% in *CCR2*^−/−^ mice that are aged between 18 and 27 months (Chen et al., 2011). Also for *Cx3cr1* deficient mice a pronounced age-related retinal degeneration has been reported. While *Cx3cr1*^*GFP/GFP*^ mice on the C57Bl/6 genetic background showed a pronounced retinal degeneration at 18 or 20 months of age respectively, the *Cx3cr1*^*GFP/GFP*^ mutation on the albino Balb/c background revealed a much greater variability for the associated retinal degeneration, with either complete light-dependent loss of photoreceptors at 4 months or with preservation of more than half of the photoreceptors at an age of 20 months (Combadiere et al., 2007; Raoul et al., 2008). Because of the discrepancies between all these reports, and because we did not observe any evidence for retinal degeneration in our related *Ccl2*^−/−^, *Cx3cr1*^−/−^ and *Ccl2*^−/−^/*Cx3cr1*^−/−^ mice on the C57Bl/6 genetic background it seems very likely that the reported retinal degeneration phenotype, as well as the associated accumulation of subretinal macrophages to a large extend are strongly influenced by additional factors. Such factors may include different ages, genetic background, or housing conditions (light, pathogen load) or the presence of unknown mutant alleles, similar to the homozygous *Crb1 rd8* mutation which is widely distributed in many transgenic lines across the world (Chinnery et al., 2012; Mattapallil et al., 2012), or unknown effects of the eGFP reporter alleles in *Cx3cr1*^*GFP/GFP*^ mice (Jung et al., 2000).

The absence of both key features, retinal degeneration and accumulation of subretinal macrophages in both these *Cx3cr1*^−/−^ deficient lines compared to the widely used *Cx3cr1*^*GFP/GFP*^ or *Cx3cr1*^*GFP/+*^ lines raises the question whether these two features are actually the consequence of the genetic defect or rather the consequence of other factors, including the long term ectopic overexpression of the reporter gene eGFP in monocytes and microglia in this line, which over long periods can be toxic for cells (Duisit et al., 2002). A prominent influence of the genetic background on subretinal microglia accumulation and retinal degeneration has already been described in different chemokine lines (Chinnery et al., 2012; Combadiere et al., 2007), but has in particular been highlighted in a model of light-induced retinal damage where healthy *C57Bl/6J-Tyr*^*c2j*^ mice, which are albino mice with an C57Bl/6-J genetic background, have significantly lower subretinal microglia than two albino strains (Balb/c/A/J) (Ng and Streilein, 2001). Alternatively, it could be that the increased C57Bl/6 genetic background in our related chemokine knockout mouse lines suppresses the manifestation of the phenotype of the respective chemokine deficiencies. This would be consistent with our recent report, in which we have observed a suppressive effect of the C57Bl/6J Ola Hsd genetic background on the manifestation of the retinal degeneration phenotype caused by the *Crb1 rd8* mutation (Luhmann et al., 2012). Similarly, it is well known that the development of ocular pathology in the experimental autoimmune uveitis (EAU) model is very much dependent on the underlying genetic background (Caspi et al., 1992). B10.RIII mice are a highly susceptible strain, whilst C57Bl/6 mice are much less sensitive to the induction of EAU.

### 4.4 Chemokine signalling defects may modulate retinal degeneration

The presence of additional endogenous or exogenous (trigger) factors might be necessary for manifestation of chemokine signalling pathway dysfunction. This is supported by studies in which enhanced neuronal cell death and microglial activation was observed in *Cx3cr1*^*GFP/GFP*^ mice following induction by peripheral injection of LPS (Cardona et al., 2006). A similar ‘priming effect’ has been suggested for the Ccl2-Ccr2 signalling pathway. Transgenic overexpression of CCL2 in astrocytes only leads to neurological impairment after systemic injection of pertussis (Huang et al., 2002). It has also been shown that photoreceptor loss after retinal detachment or light-induced degeneration can be attenuated by ablation of CCL2 or CCR2 signalling by reducing recruitment of ED1 and Iba1 positive cells (Nakazawa et al., 2007; Rutar et al., 2012). It is tempting to speculate that increased local levels of CCL2 observed during ageing (Chen et al., 2008) and in AMD patients (Jonas et al., 2010) promote retinal degeneration, not directly, but by priming the retina for the infiltration of systemic pro-inflammatory monocytes.

## Disclosure statement

The authors declare no conflict of interest.

## Funding

This project was supported by the Wellcome Trust (WT074617). The funders had no role in study design, data collection and analysis, decision to publish or preparation of the manuscript.
